# Conjugation of plasmid harboring *bla*_NDM-1_ in a clinical *Providencia rettgeri* strain through the formation of a fusion plasmid

**DOI:** 10.3389/fmicb.2022.1071385

**Published:** 2023-01-04

**Authors:** Meng Zhang, Yanhua Yu, Qian Wang, Ran Chen, Yueling Wang, Yuanyuan Bai, Zhen Song, Xinglun Lu, Yingying Hao

**Affiliations:** ^1^Department of Clinical Laboratory, Shandong Provincial Hospital, Cheeloo College of Medicine, Shandong University, Jinan, Shandong, China; ^2^Department of Clinical Laboratory, Liaocheng Second People’s Hospital, Liaocheng, Shandong, China; ^3^Department of Clinical Laboratory, Beijing Youan Hospital, Capital Medical University, Beijing, China; ^4^Department of Clinical Laboratory, Shandong Provincial Hospital Affiliated to Shandong First Medical University, Jinan, China

**Keywords:** *Providencia rettgeri*, *bla*_NDM-1_, *bla*_OXA-10_, *bla*_PER-4_, class-1 integrons (*IntI*), *IS26*, *ISKox2*

## Abstract

*Providencia rettgeri* has recently gained increased importance owing to the New Delhi metallo-β-lactamase (NDM) and other β-lactamases produced by its clinical isolates. These enzymes reduce the efficiency of antimicrobial therapy. Herein, we reported the findings of whole-genome sequence analysis and a comprehensive pan-genome analysis performed on a multidrug-resistant *P*. *rettgeri* 18004577 clinical strain recovered from the urine of a hospitalized patient in Shandong, China, in 2018. *Providencia rettgeri* 18004577 was found to have a genome assembly size of 4.6 Mb with a G + C content of 41%; a circular plasmid p18004577_NDM of 273.3 Kb, harboring an accessory multidrug-resistant region; and a circular, stable IncT plasmid p18004577_Rts of 146.2 Kb. Additionally, various resistance genes were identified in its genome, including *bla*_NDM-1_, *bla*_OXA-10_, *bla*_PER-4_, *aph*(*3′*)*-VI*, *ant*(*2′′*)*-Ia*, *ant*(*3′*)*-Ia*, *sul1*, *catB8*, *catA1*, *mph*(*E*), and *tet*. Conjugation experiments and whole-genome sequencing revealed that the *bla*_NDM-1_ gene could be transferred to the transconjugant *via* the formation of pJ18004577_NDM, a novel hybrid plasmid. Based on the genetic comparison, the main possible formation process for pJ18004577_*NDM* was the insertion of the [*ΔISKox2-IS26-ΔISKox2*]*-aph*(*3′*)*-VI-bla*_NDM-1_ translocatable unit module from p18004577_*NDM* into plasmid p18004577_Rts in the Russian doll insertion structure (*ΔISKox2-IS26-ΔISKox2*), which played a role similar to that of *IS26* using the “copy-in” route in the mobilization of [*aph*(*3′*)*-VI*]*-bla*_NDM-1_. The array, multiplicity, and diversity of the resistance and virulence genes in this strain necessitate stringent infection control, antibiotic stewardship, and periodic resistance surveillance/monitoring policies to preempt further horizontal and vertical spread of the resistance genes. Roary analysis based on 30 *P*. *rettgeri strains* pan genome identified 415 core, 756 soft core, 5,744 shell, and 12,967 cloud genes, highlighting the “close” nature of *P*. *rettgeri* pan-genome. After a comprehensive pan-genome analysis, representative biological information was revealed that included phylogenetic distances, presence or absence of genes across the *P*. *rettgeri* bacteria clade, and functional distribution of proteins. Moreover, pan-genome analysis has been shown to be an effective approach to better understand *P*. *rettgeri* bacteria because it helps develop various tailored therapeutic strategies based on their biological similarities and differences.

## 1. Introduction

*Providencia rettgeri* is an opportunistic human pathogen that belongs to the genus *Providencia*, family Morganellaceae, and order Enterobacterales. It is mainly associated with hospital-acquired infections, including catheter-related urinary tract infections, bacteremia, meningitis, diarrhea, endocarditis, and wound and eye infections (Abdallah and Balshi, 2018). *Providencia rettgeri* exhibits intrinsic resistance to many antimicrobials, including ampicillin, first-generation cephalosporins, polymyxins, and tigecycline (Shin et al., 2018), which makes the treatment of infections caused by this pathogen challenging.

Since the first carbapenem-resistant *Providencia* strain was reported in Japan in 2003 (Shibata et al., 2003), *P*. *rettgeri* became extremely popular as a carbapenemase producer; the production of metallo-β-lactamase (MBL) carbapenemases, *NDM-1* being the most common, by *P*. *rettgeri* has gained extensive attention. This indicated that the clinically available β-lactamase inhibitors, including avibactam, relebactam, and vaborbactam, were not effective against infections caused by carbapenem-resistant *Providencia* strains. Moreover, the emergence of multidrug-resistant *P*. *rettgeri* strains poses a serious threat to public health.

Horizontal gene transfer remains the most effective means of bacterial evolution, allowing bacteria to rapidly acquire new functional genes, including virulence and antibiotic resistance (AR) genes, with the help of mobile genetic elements (MGEs). These MGEs include a series of insertion sequences (ISs), plasmids, prophages, and viruses (Zhong et al., 2019). In recent years, the *IS6/IS26* family of ISs was reported to form cointegrates *via* both, the copy-in and targeted conservative mechanisms (Harmer and Hall, 2020). However, it remains unknown whether other ISs can perform the same reaction. In particular, the role of MGEs in the dissemination of *bla*_NDM-1_ among *P*. *rettgeri* strains is not well established.

In this study, we detected a *bla*_NDM-1_-producing *P*. *rettgeri* isolate in a patient with urinary tract infection at a teaching hospital in Shandong, China. Comparative genomic analyses of the plasmids harboring *bla*_NDM-1_, *bla*_OXA-10_, and *bla*_PER-4_ were conducted to elucidate the genetic environment and recombination during conjugation. Two copies of the same IS could form a composite transposon capable of mobilizing the intervening components at multiple nested genetic levels, analogous to a Russian doll set, drives rapid dissemination of the carbapenem resistance genes (Feng et al., 2015; Sheppard et al., 2016). To the best of our knowledge, this is the first report that describes a nested insertion structure *ΔISKox2-IS26-ΔISKox2*, playing a role similar to that of the *IS26* isoform in horizontal gene transfer progression.

The pan-genome has become crucial for understanding species diversity and evolution. The pan-genome refers to a complete set of genes present in a collection of organisms. Pan-genome is divided into core and accessory genomes (Tettelin et al., 2005). The core genome is likely essential for the growth or survival of the clade whereas the accessory genome is considered to be composed of major genes to understand variations in the clade’s genomes and their specific lifestyles and evolutionary trajectories (Kim et al., 2020).

In this study, a comprehensive analysis of *P*. *rettgeri* strain genomes, by means of pan-genome analysis, both at the phylogenetical and functional level, may provide useful insights into the different properties of *P*. *rettgeri* strains.

## 2. Materials and methods

### 2.1. Clinical case, clinical strain, and susceptibility assays

Carbapenem-resistant *P*. *rettgeri* strain 18004577 was recovered from the urine sample of an 18-year-old male patient with chest and abdominal trauma who was admitted to Shandong Provincial Hospital, China, in 2018. The patient was diagnosed with multiple fractures and lung and kidney contusions after falling from a six-story building. Urinary tract infection occurred during hospitalization. Finally, the patient recovered and was discharged. The patient’s medication history and hospital course details were retrieved from the hospital record information system. The case history collection and reporting protocols used in the present study were approved by the Ethics Committee of Shandong Provincial Hospital.

Species identification was performed using matrix-assisted laser desorption/ionization time-of-flight mass spectrometry (BioMérieux, France). Phenotypic detection of carbapenemases was conducted using the carbapenem inactivation method (CIM) and the EDTA-modified CIM (eCIM) test, according to the guidelines of the Clinical and Laboratory Standards Institute (CLSI). Antibiotic susceptibility testing for aztreonam, cefepime, ceftriaxone, ceftazidime, ertapenem, imipenem, piperacillin/tazobactam, trimethoprim/sulfamethoxazole, ciprofloxacin, levofloxacin, gentamicin, amikacin, ampicillin, ampicillin–sulbactam, cefazolin, cefotetan, tobramycin, and nitrofurantoin was conducted using the VITEK-2 compact system (BioMérieux, France); the test results were interpreted using CLSI breakpoints (CLSI, 2020).

### 2.2. Conjugation assay and confirmation of the locations of the resistance genes

Conjugation experiments were conducted as described previously, with sodium azide-resistant *E*.*coli* J53AziR as the recipient strain and *P*. *rettgeri* 18004577 as the donor strain. Transconjugants harboring carbapenemase resistance genes were selected on Mueller–Hinton agar plates containing 6 μg/ml ceftazidime and 100 mg/ml sodium azide. Antibiotic susceptibility tests and PCR were performed to confirm carbapenemase gene transfer (Poirel et al., 2011; Xiang et al., 2015).

S1-nuclease pulsed-field gel electrophoresis (S1-PFGE) was performed to determine the size and number of plasmids carried by *P*. *rettgeri* 18004577 (clinical strain) and J-18004577, a transconjugant. The genomic DNA of the clinical strain and transconjugant embedded in the gel plugs was digested with QuickCut S1 nuclease (Takara, Shiga, Japan) for 1 h or with QuickCut sfi nuclease (Takara, Shiga, Japan) for 2.5 h and then separated using S1-PFGE for 17 h. The pulse time was switched from 2.16 to 63.8 s. *Salmonella* strain H9812 was digested with QuickCut XbaI (Takara, Shiga, Japan) and used as the reference marker. Southern blotting was conducted to confirm the locations of blaNDM−1, blaOXA-10, and blaPER-4 using specific probes labeled with digoxigenin (Roche, Basel, Switzerland; Supplementary Figure 1A) A 621-bp probe for blaNDM-1 was synthesized using PCR amplification with the primers 5′-CGGAATGGCTCATCACGATCCAC-3′ (forward) and 5′-GGTTTGGCGATCTGGTTTTC-3′ (reverse). A 504-bp probe for blaOXA-10 was synthesized using the primers 5′-TCTGCCGAAGCCGTCAATGGT-3′ (forward) and 5′-ATATTCAGGTGCCGCCTCCGTTA-3′ (reverse). Finally, a 504-bp probe for blaPER-4 was synthesized using the primers 5`-GCAATACTCGGTCTCGCACAC′-3′ (forward) and 5′-TGATACGCAGTCTGAGCAACCT-3′ (reverse).

### 2.3. Whole genome sequencing and annotation

DNA was extracted from the clinical isolates and transformants using a genomic DNA commercial kit (Qiagen, Hilden, Germany). Genomic DNA was sequenced using the Illumina HiSeq platform (Novogene Co., Ltd., Beijing, China) and PacBio RSII sequencer (Biozeron Biological Technology Co., Ltd., Shanghai, China). Paired-end short Illumina reads were used to correct long PacBio reads using proofread for large-scale high-accuracy PacBio correction, and the corrected PacBio reads were then assembled *de novo* utilizing using the functions available at https://github.com/ruanjue/smartdenovo.

Sequence annotation was conducted using RAST 2.0^1^ combined with BLASTP/BLASTN searches against UniProtKB/SwissProt and RefSeq databases. Annotation of the resistance genes and mobile elements was conducted using online databases, including CARD^2^ and ISfinder.^3^ PHAge Search Tool Enhanced Release was used to identify prophages (Arndt et al., 2016). The virulence genes were identified by alignment of the gene sequences against the sequences available in the Virulence Factors Database (Liu et al., 2019).

### 2.4. Comparison analysis of plasmid sequences

The sequences of five plasmids were retrieved from the NCBI database for comparative analysis: pBML2531 (GenBank accession no. AP022376.1), pNDM15-1091 (GenBank accession no.CP012903.1), pRts1 (GenBank accession no. MN626604.1), pT-OXA-181 (GenBank accession no. NC_020123.1), and Rts1 (GenBank accession no. NC_003905.1).

A BLAST Ring Image Generator^4^ was used to generate and visualize the comparisons of the plasmids and their genetic structures. More detailed genome alignment between closely related plasmids was conducted using local BLAST, and the findings were visualized using Easyfig.^5^

### 2.5. Plasmids stability testing

Plasmid stability testing was performed using Luria–Bertani broth as previously described, but with some modifications (Nang et al., 2018). Briefly, the *P*. *rettgeri* 18004577 clinical strain was cultured at 37°C in a shaking bath (150 rpm/min) and serially passaged for 7 days with a 1:1,000 dilution of antibiotic-free Luria–Bertani broth. Then, 100 μl of the seventh-day culture was plated onto antibiotic-free Mueller Hinton agar. Subsequently, 50 colonies were randomly selected and subjected to PCR for amplification of the *repA* gene of the plasmid p18004577_Rts. The plasmids were considered stable if more than 85% of the colonies harbored the repA gene.

### 2.6. Pan-genome analysis of the reported *Providencia rettgeri* strains

Combining with sequencing data of 29 published *Providencia rettgeri* genomes from the National Center for Biotechnology Information (NCBI) database, pan-genome analysis was carried out (Supplementary Table 1). The criteria for data-selection was that *Providencia rettgeri* strain with select columns “level” had “complete” genome files available on NCBI in the beginning of our project. We first determined the phylogenetic relationship between the 30 *P*. *rettgeri* strains using OrthoFinder (Emms and Kelly, 2015), by using the protein sequences of the strains obtained from the Prokaryotic Genome Annotation System (Prokka; Seemann, 2014). Further, the multiple sequence alignment analysis of the resulting single-copy direct-line homologous protein long sequence was carried out using the MAFFT software (Katoh, 2005). Then, we constructed the maximum-likelihood phylogenetic tree of 30 genomes using the RaxML-NG software, with a 1,000-bootstrap test (Kozlov et al., 2019). The calculation of the best amino-acid substitution model was performed using ModelTest-NG software before the phylogenetic tree construction (Darriba et al., 2020). After obtaining the best tree, we visualized the evolutionary tree using the R software with ggtree package (Yu, 2020). Average nucleotide identity (ANI) was performed to explore the taxonomic boundary of the genomes using FastANI (Jain et al., 2018).

After the determination of potential confounding strains, pan-genome analysis was conducted with Roary (Page et al., 2015) using the GFF3 files generated by Prokka (Seemann, 2014). To explore the molecular and biological functions of core genes from the 30 genomes, GO functional enrichment analysis was carried using the R language clusterProfiler package (Wu et al., 2021).

## 3. Results

### 3.1. Overview of the *Providencia rettgeri* clinical isolate

Carbapenem-resistant *P*. *rettgeri* 18004577 was identified as an MBL-producing strain using eCIM. The antimicrobial susceptibility testing results are presented in Table 1. The clinical strain was resistant to almost all of the 18 antibiotics, except for amikacin and trimethoprim/sulfamethoxazole. The transconjugant J-18004577 remained susceptible to amikacin, trimethoprim/sulfamethoxazole, aztreonam, and nitrofurantoin.

**Table 1:** Antibiotic susceptibility testing of *Providencia rettgeri* 18004577 and the transconjugant J-18004577.

Strains	MIC (μg/ml)																	
	AMP	SAM	TZP	CZO	CTT	CRO	CAZ	FEP	ATM	ETP	IMP	AMK	CEN	TOB	CIP	LVX	FIT	SXT
*P*. *rettgri*	≥32 (R)	≥32 (R)	64 (R)	≥64 (R)	≥64 (R)	≥64 (R)	≥64 (R)	≥64 (R)	≥64 (R)	≥8 (R)	≥16 (R)	4 (S)	8 (I)	8 (I)	≥4 (R)	4 (I)	128 (R)	≤20 (S)
1.8E+07																		
Transconjugant	≥32 (R)	≥32 (R)	≥128 (R)	≥64 (R)	≥64 (R)	≥64 (R)	≥64 (R)	≥64 (R)	≤1 (S)	≥8 (R)	≥16 (R)	≤2 (S)	≤1 (S)	≤1 (S)	≤0.25 (S)	≤0.25 (S)	≤16 (S)	≤20 (S)
J-18004577																		

MIC, Minimal inhibitory concentrations; AMP, ampicillin; SAM, ampicillin/sulbactam; TZP, piperacillin/tazobactam; CZO, cefazolin; CTT, cefotetan; CRO, cefatriaxone; CAZ, ceftazidime; FEP, cefepime; ATM, aztreonam; ETP, ertapenem; IMP, imipenem; AMK, amikacin; CEN, gentamicin; TOB, tobramycin; CIP, ciprfloxacin; LVX, levofloxacin; FIT, nitrofurantion; and SXT, trimethoprim/sulfamethoxazole.

According to the whole-genome sequencing analysis, the complete genome of strain 18004577 contained a circular chromosome of 4.6 Mb with a G + C content of 41%, a circular plasmid p18004577_NDM of 273.2 Kb with a G + C content of 47.6%, and a circular plasmid p18004577_Rts of 146.2 Kb with a G + C content of 45.5%.

A total of 10 prophage regions were identified, of which eight were located on the genome and two were located on the plasmid p18004577_NDM (Supplementary Table 2). A CRISPR region was identified in the genome of 18004577 at nucleotide positions 565, 107–565, 213-bp, with one 29-bp long spacer sequence.

### 3.2. Distribution of virulence genes

The distribution of virulence genes was investigated to identify the key pathogenicity genes of *P*. *rettgeri* 18004577. The strain harbored several virulence genes, such as *flhA*, *fliC*, *clpB*, *hcp*, *fcl*, and *gmd*, associated with flagellar biosynthesis, type VI secretion system, and O-antigen (Supplementary Table 3).

### 3.3. Characteristics of plasmids carrying *bla*_NDM-1_ in the clinical strain and transconjugant

According to the results of S1-PFGE, the clinical strain *P*. *rettgeri* 18004577 harbored two plasmids of approximately 300 and 100 Kb (Supplementary Figure 1B). Based on the whole-genome analysis, the larger plasmid named p18004577_NDM (GenBank accession no. CP098041.1) was found to harbor multiple resistance genes, including *bla*_NDM-1_, *bla*_OXA-10_, *bla*_PER-4_, *aph*(*3′*)*-VI* (kanamycin resistance), *ant*(*2′′*)*-Ia* (gentamicin and tobramycin resistance), *ant*(*3′*)*-IIa* (streptomycin resistance), *sul1* (sulfamethoxazole resistance), *catB8* and *catA1* (chloramphenicol resistance), *mph*(*E*; erythromycin resistance), and *tet* (tetracycline resistance; Supplementary Table 4).

**Figure 1:**
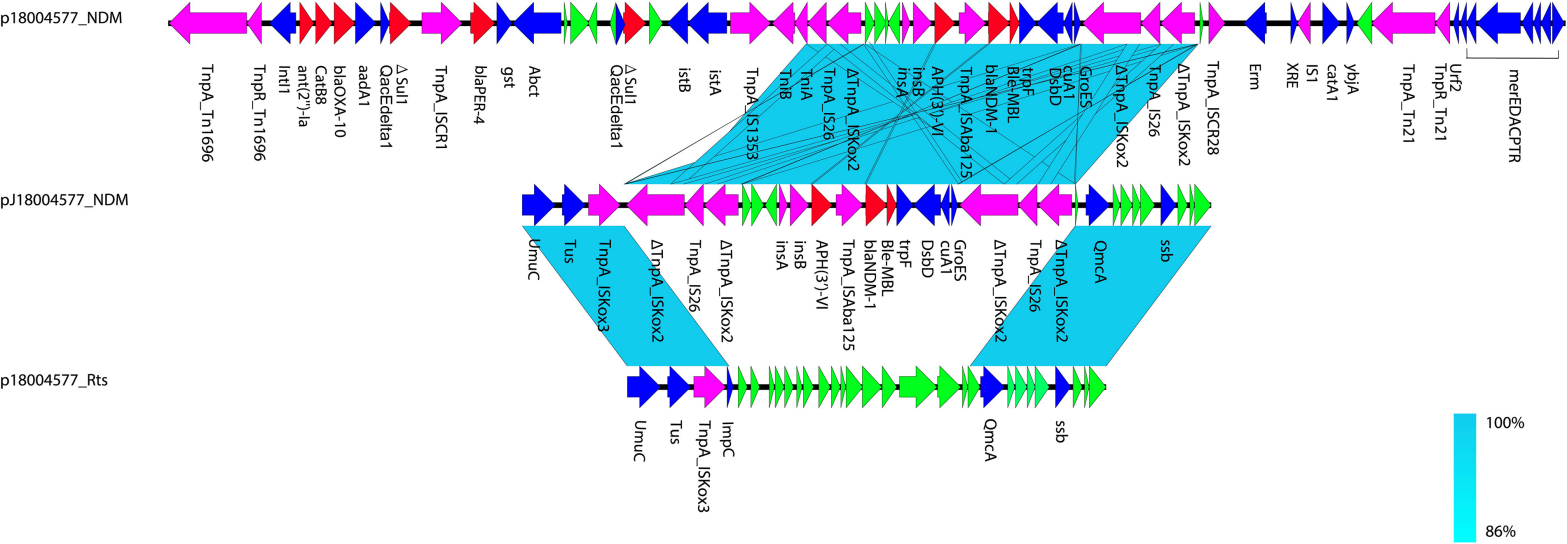
Comparison of p18004577_NDM, pJ18004577_NDM, and p18004577_Rts. Colors of the arrows indicate the functions of the predicted ORFs: accessory genes (blue), antimicrobial resistance genes (red), transposon-related genes and insertion sequences (purple), and hypothetical proteins (green). Regions with homology are shaded light blue.

The New Delhi MBL (NDM)-harboring plasmid of the clinical strain was transferred into *E*. *coli* J53Azi^R^ *via* conjugation. The presence of NDM-1 in the transconjugant was confirmed using PCR. Two resistance genes were identified in the transconjugant J-18004577––*bla*_NDM-1_ and *aph*(*3′*)*-VI*––which were obtained from the clinical isolate *P*. *rettgeri* 18004577 by conjugation assay (Figure 1). Southern blotting confirmed that *bla*_NDM-1_, *bla*_OXA-10_, and *bla*_PER-4_ were located on the larger plasmid in the clinical strain, and only *bla*_NDM-1_ was present on the hybrid plasmid, with a size of approximately 220 Kb of the transconjugant (Supplementary Figures 1C,D).

**Figure 2:**
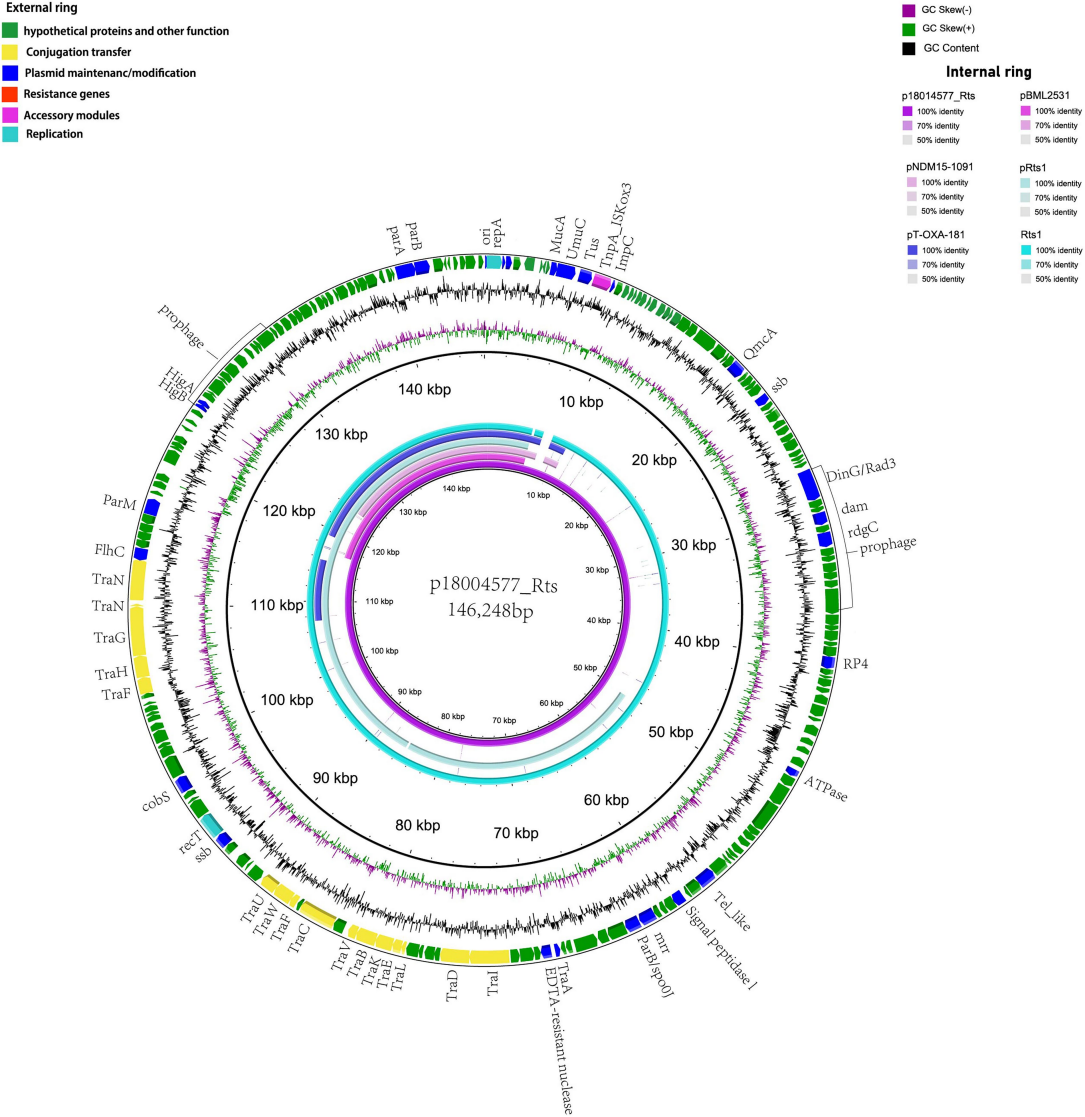
Structural comparisons of p18004577_Rts with the similar plasmids. The outer ring represents the CDSs of the reference sequence p18004577_Rts. The internal six rings show the comparative analysis of plasmids pBML2531 (GenBank accession no. AP022376.1), pNDM15-1091 (GenBank accession no.CP012903.1), pRts1 (GenBank accession no. MN626604.1), pT-OXA-181 (GenBank accession no. NC_020123.1), and Rts1 (GenBank accession no. NC_003905.1) with p18004577_Rts.

p18004577_Rts was found to be a type IncT plasmid with a 146,248-bp size and an average GC content of 45.5%; it had at least 202 predicted coding sequences. No resistance gene was predicted, and it included 15 tra-type genes, except for *trb*, in the transfer region. BLAST analysis showed that p18004577_Rts shared 99% nucleotide identity with 98% cover with plasmid Rts1 from *Proteus vulgaris* (NC_003905.1; Figure 2).

p18004577_Rts (GenBank accession no. CP098042.1) harbored by *P*. *rettgeri* 18004577 contained a replication initiation protein encoded by *repA* (at 155–1177 bp) and a short segment containing the replication origin ori (at 51–238 bp) that provides two elements for its autonomous replication (Murata et al., 2002), a process which is similar to the one found in plasmid Rts1. The replication of this mini-Rts1 plasmid was stable at 37°C (Itoh et al., 1982) The stability of p18004577_Rts was confirmed using PCR amplification performed after 7 days of culture; 48 of 50 colonies were found to carry *repA*, indicating the high stability of p18004577_Rts.

**Figure 3:**
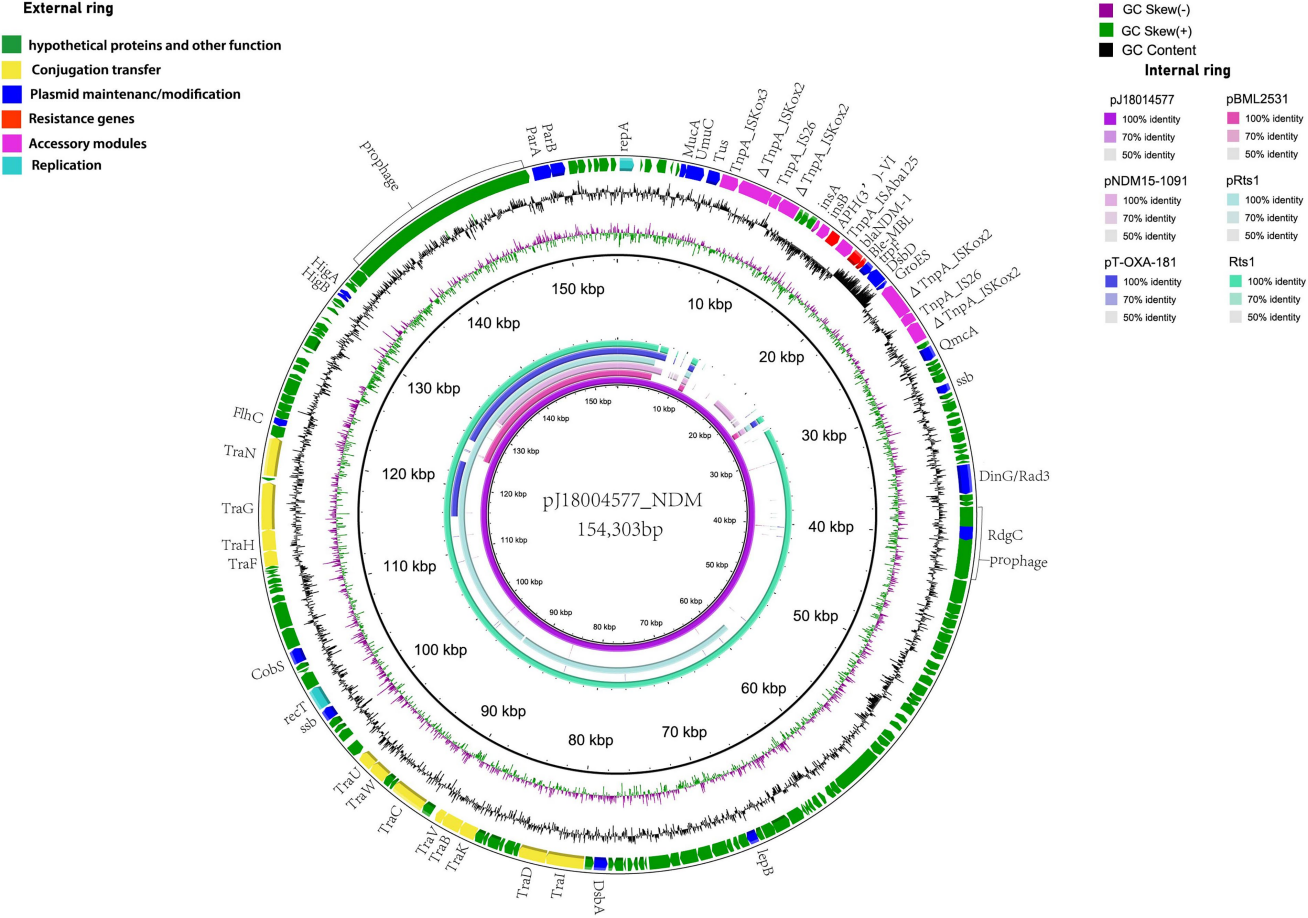
Comparisons of genetic context of pJ18004577_NDM with similar plasmids. Red and purple arrows represent antibiotic resistance genes and accessory modules, respectively. The features of the variable region (at 8,779–24,607 bp) shared high homology with the *bla*_NDM-1_-containing region of p18004577_NDM, and the backbone region was almost identical to that of p18004577_Rts.

pJ18004577_NDM (GenBank accession no. CP114206) was a hybrid plasmid generated from p18004577_NDM to p18004577_Rts, with a length of 154,303 bp, a GC content of 45.5%, and 209 predicted coding sequences. The features of the variable region (at 8,779–24,607 bp) of pJ18004577_NDM shared high homology with the *bla*_NDM-1_-containing region of p18004577_NDM, and the backbone region of pJ18004577_NDM was almost identical to that of p18004577_Rts (Figure 3).

### 3.4. Comparative analysis of the genetic environment of blaNDM-1

**Figure 4:**
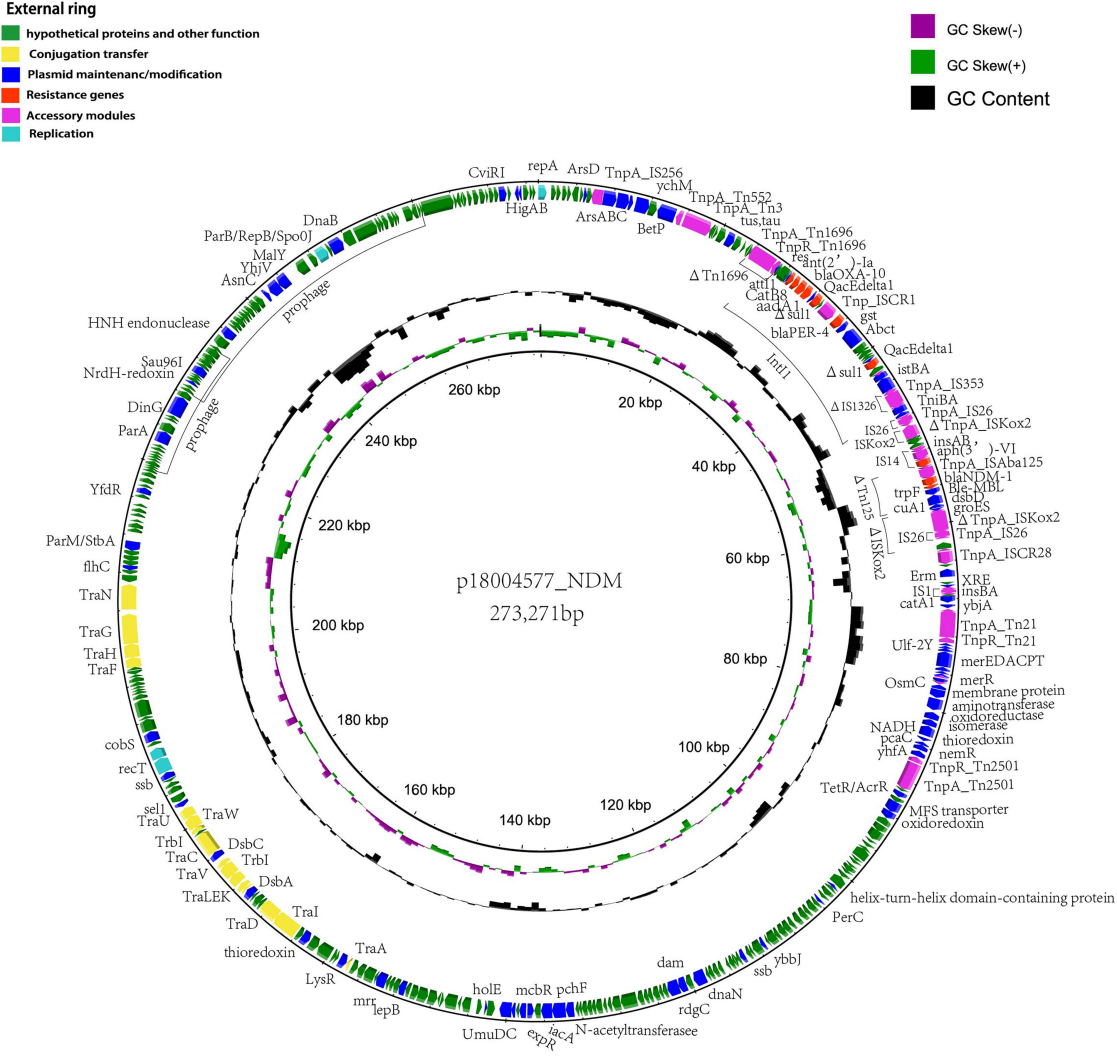
Schematic representation of the genetic organization of p18004577_NDM. The outermost ring shows the coding sequences of the plasmid. Each coding sequence is color coded according to its predicted function, as shown in the figure legend. The two inner rings represent the GC content and the GC skew graph, respectively.

According to the whole-genome analysis, p18004577_NDM harboring *bla*_NDM-1_ from clinical strain *P*. *rettgeri* 18004577 was a 273,271-bp long untypeable plasmid, based on replication module analysis. p18004577_NDM contained an accessory multidrug-resistant region generated by *ΔTn1696*, class 1 integrons (*IntI*) harboring *bla*_OXA-10_, *ant* (*2′*)*-Ia*, *catB8*, *bla*_PER-4_ and *sul*, *ΔIS1326*, *ΔTn125* harboring *bla*_NDM-1_, *ΔISKoX2*, *ΔTn21*, *mer* operon, and *ΔTn2501*, which conferred resistance to aminoglycosides, quinolones, and β-lactams. In addition, two prophage regions (at 218,975–233,789 and 288,401–260,814 bp) were predicted, the physiological functions of which are unknown (Figure 4).

**Figure 5:**
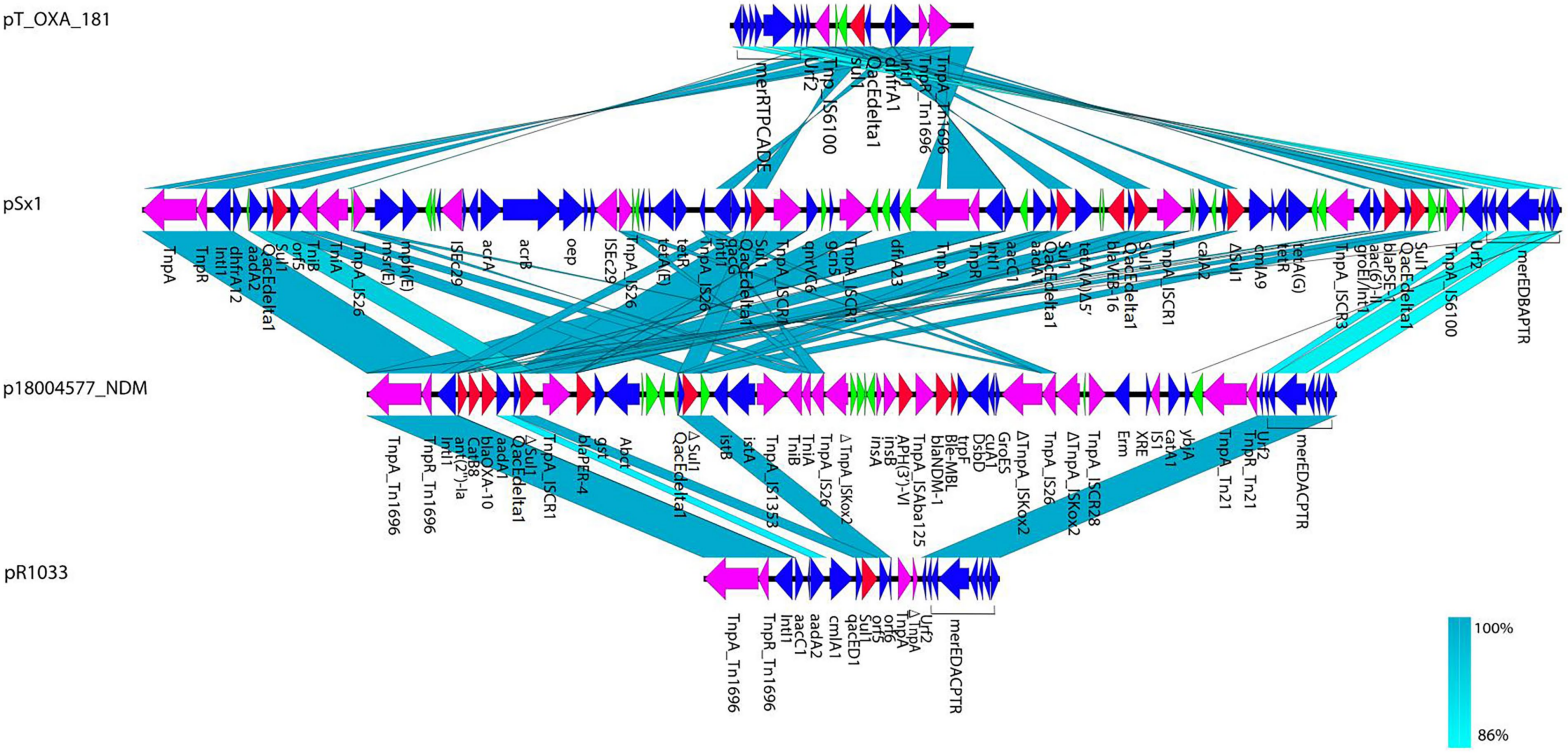
Structural comparisons of Tn1696 from p18004577_NDM, pSx1 (accession no. CP013115), pT_OXA_181 (accession no. JQ996150), and pR1033 (accession no. U12338). Arrows indicate the deduced open reading frames (ORFs) and their orientations. Blue arrows were used for ORFs encoding plasmid basic functions and green arrows expressing hypothetical proteins. Resistance genes are indicated by red arrows, while transposon-related genes (tnpA and tnpR) and insertion sequences are indicated by purple arrows.

In p18004577_NDM, a variant of *Tn1696* was observed upstream of the class I integron, which was found to be similar to pSx1 (accession no. CP013115), pT_OXA_181 (accession no. JQ996150), and *Tn1696* (accession no. U12338; Figure 5). The class I integron (at 27,283–48,006 bp) was flanked by an *IS26* sequence that interrupted the transposase *TniA* at a downstream position, and the cassette content of the class I integron comprised multiple resistance genes, such as *bla*_OXA-10_, *ant* (*2′*)*-Ia*, *catB8*, *bla*_PER-4_, two *Δsul1*, two *qacEdelta1*, and one *IS1326* element truncated by *IS1353*. The *bla*_NDM-1_ containing region (at 53,799–58,491 bp) was truncated by *Tn125* that was flanked by an *IS14* element at an upstream location and by *ISKoX2* inserted by *IS26* at a downstream location (Figures 1, 4).

The plasmid pJ18004577_NDM showed partial homology with several other plasmids, such as pBML2531, pNDM15-1,091, pRts1, pT-OXA-181, and Rts1 (Figure 3). The features of the variable region (at 8,779–24,607 bp) of pJ18004577_NDM shared high homology with the *bla*_NDM-1_-containing region of p18004577_NDM, and the backbone region of pJ18004577_NDM was highly similar to that of p18004577_Rts.

**Figure 6:**
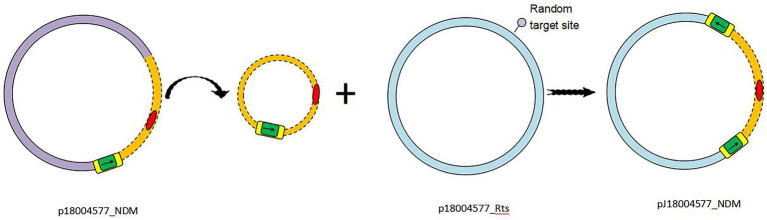
Schematic of transconjugant formation. IS26 is shaded green, with the left and right ΔISKox2 shaded yellow. Antibiotic resistance genes are colored red. The p18004577_NDM backbone is colored light purple, whereas the p18004577_Rts backbone is light blue. Small black arrows indicate the orientation of the IS26 and ΔISKox2 genes.

Based on the results of genetic comparison, the main possible formation process of pJ18004577_NDM was the insertion of the [*ΔISKox2-IS26-ΔISKox2*]*-*[*aph*(*3′*)*-VI*]*-bla*_NDM-1_ module from p18004577_NDM (at 48,007–63,004 bp) into plasmid p18004577_Rts (at 8,779–17,992 bp; Figure 1). The insertion module in pJ18004577_NDM seemed to be surrounded by an 8-bp target site duplication with the sequence “GCTGAGAT” (at 8,871–8,878 bp) and “TTGAAGAA” (at 13,292–13,299 bp) at random sites; this may have been due to a cointegrate (Figure 6).

### 3.5. Main genomic features of *Providencia rettgeri* pan-genome

OrthoFinder assigned 123,540 genes (98.3% of the total) to 6,649 orthogroups. Fifty percent of all genes were in orthogroups with 30 or more genes (G50 was 30) and were contained in the largest 2042 orthogroups (O50 was 2042). There were 2,591 orthogroups with all species present and 2,442 of these consisted entirely of single-copy genes.

**Figure 7:**
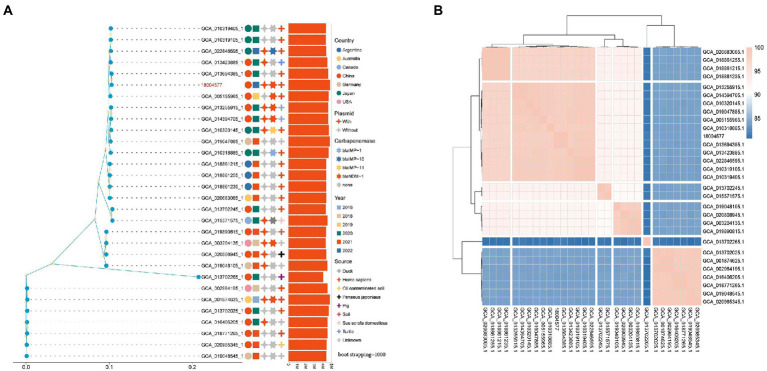
**(A)** Phylogenetic tree of the *Providencia rettgeri* strains. Isolation source, year, and geographical provenience of each strain are reported. The number of plasmids and types of carbapenemase genes were marked with different colors and shapes on the right side of the tree; **(B)** Correlation matrix of average nucleotide identity for the 30 *P*. *rettgeri* genomes obtained by FastANI.

The maximum-likelihood phylogenetic tree constructed based on the best amino acid substitution model of 30 genomes single-copy lineal homolog proteins was used to further explore genomic similarities among strains showed in Figure 7A. The analysis of 30 *P*. *rettgeri* genomes by FastANI showed that higher the association between the microbial species, the higher the ANI values between each other (Figure 7B). The ML phylogenetic tree revealed that strains could be broadly clustered into two major clades base on the genetic distance calculated by the ANI value. The sample sources were scattered among the two clades of phylogenetic trees.

**Figure 8:**
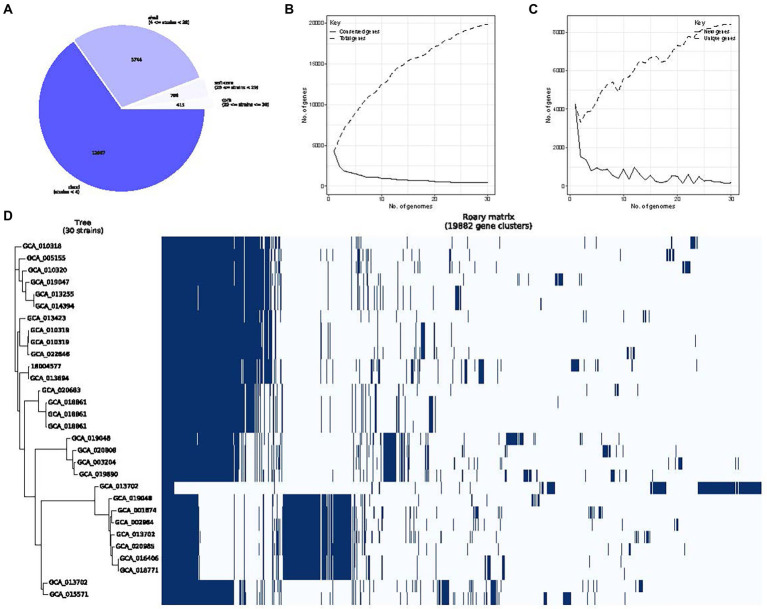
*Providencia rettgeri* pan-genome. **(A)** The number of genes belonging to the core, soft core, shell or cloud of the *P*. *rettgeri* species is represented as a pie chart; **(B)** Conserved genes frequency vs. genome number; **(C)** Representation of *P*. *rettgeri* gene content according to the pan-genome variation since the genomes are added in random order to the analysis. The short line represents unique genes; the long line represents new genes; and **(D)** The gene presence-absence profile of *P*. *rettgeri* strains.

Roary analysis of *P*. *rettgeri* pan-genome identified 415 core, 756 soft core, 5,744 shell and 12,967 cloud genes. As the number of sequenced genomes increases, the species’ pan-genome size converges to a certain value, highlighting the “close” nature of *P*. *rettgeri* pan-genome. Accordingly, we obtained four different classes of genes belonging to “core” (29 ≤ strains ≤ 30), “soft core” (28 ≤ strains < 29), “shell” (4 ≤ strains < 28), and “cloud” (strains <4) groups, respectively (Figure 8A). Pan-genomics of the whole dataset was performed, followed by plotting the pan genome matrix. The matrix disclosed the deviance in the presence-absence profile of the 30 *P*. *rettgeri* strains (Figures 8B–D). The functional enrichment analysis of these 415 core genomes with the results are shown in Supplementary Figure 2.

## 4. Discussion

The first case of carbapenem-resistant *Providencia* was reported in Japan in 2003; since then, carbapenem-resistant *Providencia* has been detected in many countries (Shibata et al., 2003; Abdallah and Balshi, 2018). The first clinical isolate of NDM-1-producing *P*. *rettgeri* was reported in Israel in 2013 (Gefen-Halevi et al., 2013). The high resistance of *P*. *rettgeri* to carbapenems is associated with the production of *bla_NDM-1_*. Recently, Shen and Huang et al. reported an *NDM-1*, *VIM-1*, and *OXA-10* co-producing *P*. *rettgeri* strain P138, which has a similar drug resistance spectrum as the strain reported in our study, with resistance to imipenem and ertapenem carbapenems (Shen et al., 2021). The horizontal dissemination of resistance between bacteria can occur *via* conjugative plasmids and integrative conjugative elements (He et al., 2015). In the latest study, Watanabe and Nakano et al. investigated the genetic structure of unique plasmids harboring.

*bla*_IMP-70_ and *bla*_CTX-M-253_ in multidrug-resistant *Providencia Rettgeri* and suggested that the cointegration of plasmids in *P*. *rettgeri* may not be unusual and may play a role in the transmission of clinically relevant b-lactamases (Watanabe et al., 2022). In our study, one class 1 integron was identified in p18004577_NDM, which harbored multiple resistance genes, such as *bla*_*O*XA*-10*_, *ant* (*2′*)*-Ia*, *catB8*, *bla_PER-4_*, and two *Δsul1*.

As for insertion sequences, *IS26* elements are known to play a key role in the dissemination of antibiotic resistance genes and are often combined with *Tn125* family transposons (Poirel et al., 2011). *IS6/IS26* family ISs can form cointegrates *via* both copy-in and targeted conservative mechanisms in the recruitment and spread of antibiotic resistance genes for gram-negative bacteria. In p18004577_NDM, the *bla*_NDM-1_-containing region was truncated by *Tn125* bracketing with one copy of *ISAba125*, which is likely the original mobilization mechanism of the *en bloc* acquisition of both the *bla*_NDM-1_ and *ble*_MBL_ genes (Poirel et al., 2012). Overall, the genetic environment surrounding *bla*_NDM-1_ involves [*ΔISKox2-IS26-ΔISKox2*]*-*[*aph*(*3′*)*-VI*]*-bla*_NDM-1_, which occurs in replicative transposition, as confirmed by conjugation experiments. Interestingly, this is the first study that describes its Russian doll insertion structure [*ΔISKox2-IS26-ΔISKox2*], which plays a role similar to that of *IS26* using the “copy-in” mechanism for the mobilization of [*aph*(*3′*)*-VI*]*-bla*_NDM-1_. We tried to detect circular intermediates of [*ΔISKox2-IS26-ΔISKox2*], but did not succeed.

The prototype IncT large plasmid Rtsl was originally isolated from a clinical strain of *Pr*. *vulgaris* (Terawaki et al., 1967) and expressed pleiotropic thermosensitive phenotypes in autonomous replication (DiJoseph, 1974), conjugative transferability (Terawaki et al., 1967), host cell growth (Terawaki et al., 1967; DiJoseph et al., 1973), and T-even phage restriction (Janosi et al., 1994). In our study, the p18004577_Rst strain shared 99% nucleotide identity with 98% cover with plasmid Rts1, but its temperature-sensitive replication needs further assessment.

In this study, we relied on publicly available complete genome sequences to construct a phylogenetic tree of globally reported *P*. *rettgeri* strains. The relationship and epidemiological distribution of all of the deposited *P*. *rettgeri* genomes in GenBank are depicted in Figure 7A Interestingly, globally *bla*_NDM-1_-producing *P*. *rettgeri* isolates were mainly reported in China, whereas *bla*_IMP-_producing isolates were found in Japan (Figure 7A and Supplementary Table 1). Meanwhile, carbapenemase producers were dispersedly distributed among all *P*. *rettgeri* strains. This suggests that the subsequent evolution of carbapenemase-producing *P*. *rettgeri* strains can be mainly attributed to the acquisition of genetic material through horizontal gene transfer of mobile genetic elements during the spread of these strains globally. However, this study provides a comprehensive pan-genome analysis of *P*. *rettgeri*. The dissection of *P*. *rettgeri* pan-genome into the four different gene categories (“core,” “soft core,” “shell,” and “cloud”) will facilitate genetic engineering strategies for genomic reduction/optimization. Furthermore, understanding the origin of isolation of each strain and their niche-specific adaptation can surveillance and prevent the spread of resistant strains.

## 5. Conclusion

The pathogenicity of the multidrug-resistant *P*. *rettgeri* 18004577 strain reveals the significant mobilome of this pathogen, and the presence of resistance genes, such as *bla*_NDM-1_, *bla*_PER-4_, and *bla*_OXA-10_ contribute significantly to carbapenem resistance in *P*. *rettgeri*. Taken together, our findings enhanced the knowledge of the diversity of pathogenicity, antibiotic resistance, and mobilome of the genus *Providencia*. *Providencia rettgeri* could be reservoir carbapenemase genes and can transmit these genes to other organisms *via* horizontal gene transfer. In the future, researchers should aim to increase and enhance the monitoring of carbapenemase and perform combined antimicrobial susceptibility tests to seek an effective therapeutic regimen for infections caused by Carbapenem-resistant Enterobacteriaceae strains. The comparative genomic analysis conducted in this study provides new insights into the genomic content and variability of *P*. *rettgeri* confirming that the genomic screening of new strains is essential since the bacterial genomes are dynamic entities.

## Data availability statement

The data presented in the study are deposited in the NCBI repository, accession number CP114206.

## Ethics statement

Written informed consent was obtained from the individual(s) for the publication of any potentially identifiable images or data included in this article.

## Author contributions

YH contributed to experiment conception and design. YY and MZ conducted bioinformatics analysis and wrote the paper. YB and ZS performed data analysis. RC and YW carried out bacteria identification. XL and QW prepared the tables and figures. YH is responsible for submitting a competing interests’ statement on behalf of all authors of the paper. All authors contributed to the article and approved the submitted version.

## Funding

The study was supported by grants from National Natural Science Foundation of China (81902119) and Shandong Province Natural Science Foundation (ZR2020MH306).

## Conflict of interest

The authors declare that the research was conducted in the absence of any commercial or financial relationships that could be construed as a potential conflict of interest.

## Publisher’s note

All claims expressed in this article are solely those of the authors and do not necessarily represent those of their affiliated organizations, or those of the publisher, the editors and the reviewers. Any product that may be evaluated in this article, or claim that may be made by its manufacturer, is not guaranteed or endorsed by the publisher.

## Footnotes

^1^ https://rast.nmpdr.org/

^2^ http://arpcard.mcmaster.ca

^3^ https://www-is.biotoul.fr/

^4^ https://sourceforge.net/projects/brig/

^5^ http://mjsull.github.io/Easyfig/files.html
