# Supplementary material for: Conjugation of plasmid harboring blaNDM-1 in a clinical Providencia rettgeri strain through the formation of a fusion plasmid
Source: Front Microbiol. 2023 Jan 4;13:1071385. doi: 10.3389/fmicb.2022.1071385 (PMC9845711; doi:10.3389/fmicb.2022.1071385)
Supplement: Supplementary file 1 [file Data_Sheet_1.zip › Table 4.DOCX]

Table S4: corresponding resistance genes of *P.rettgeri* 18004577

| SEQUENCE | START | END | STRAND | GENE | %COVERAGE | %IDENTITY | DATABASE | RESISTANCE |
| --- | --- | --- | --- | --- | --- | --- | --- | --- |
| 18004577-Plasmid1 | 28644 | 29177 | + | ANT(2'')-Ia | 100 | 100 | card | aminoglycoside |
| 18004577-Plasmid1 | 29258 | 29890 | + | catB8 | 100 | 97.16 | card | phenicol |
| 18004577-Plasmid1 | 29959 | 30759 | + | OXA-10 | 100 | 100 | card | cephalosporin;penam |
| 18004577-Plasmid1 | 30756 | 31567 | + | ANT(3'')-IIa | 83.54 | 99.75 | card | aminoglycoside |
| 18004577-Plasmid1 | 32072 | 32911 | + | sul1 | 100 | 100 | card | sulfonamide |
| 18004577-Plasmid1 | 35180 | 36106 | + | PER-4 | 100 | 100 | card | carbapenem;cephalosporin;monobactam;penam;penem |
| 18004577-Plasmid1 | 41052 | 41891 | + | sul1 | 100 | 100 | card | sulfonamide |
| 18004577-Plasmid1 | 52925 | 53704 | + | APH(3')-VI | 100 | 100 | card | aminoglycoside |
| 18004577-Plasmid1 | 54982 | 55794 | + | NDM-1 | 100 | 100 | card | carbapenem;cephalosporin;cephamycin;penam |
| 18004577-Plasmid1 | 55798 | 56163 | + | determinant_of_bleomycin_resistance | 100 | 100 | card | glycopeptide |
| 18004577-Plasmid1 | 67762 | 68421 | + | catI | 100 | 99.85 | card | phenicol |
| 18004577-genome | 2416258 | 2416890 | - | CRP | 100 | 81.36 | card | fluoroquinolone;macrolide;penam |
| 18004577-genome | 3065685 | 3066560 | - | CTX-M-3 | 100 | 100 | card | cephalosporin |
| 18004577-genome | 3081396 | 3082280 | - | mphE | 100 | 100 | card | macrolide |
| 18004577-genome | 3082336 | 3083811 | - | msrE | 100 | 100 | card | lincosamide;macrolide;oxazolidinone;phenicol;pleuromutilin;streptogramin;tetracycline |
