# Supplementary figures and images for: Conjugation of plasmid harboring blaNDM-1 in a clinical Providencia rettgeri strain through the formation of a fusion plasmid
Source: Front Microbiol. 2023 Jan 4;13:1071385. doi: 10.3389/fmicb.2022.1071385 (PMC9845711; doi:10.3389/fmicb.2022.1071385)

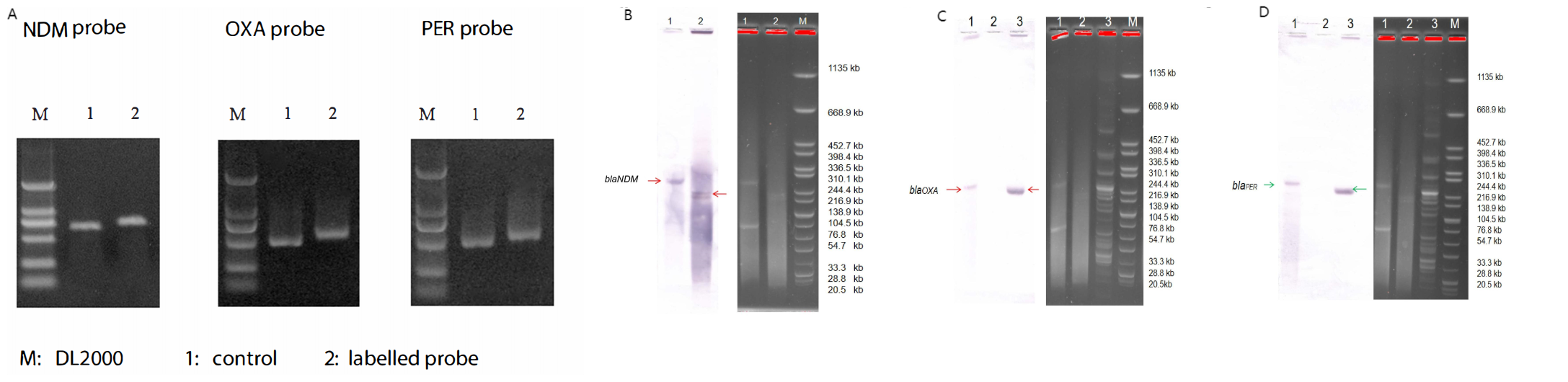

Supplement: Supplementary file 2 [file Image_1.png]

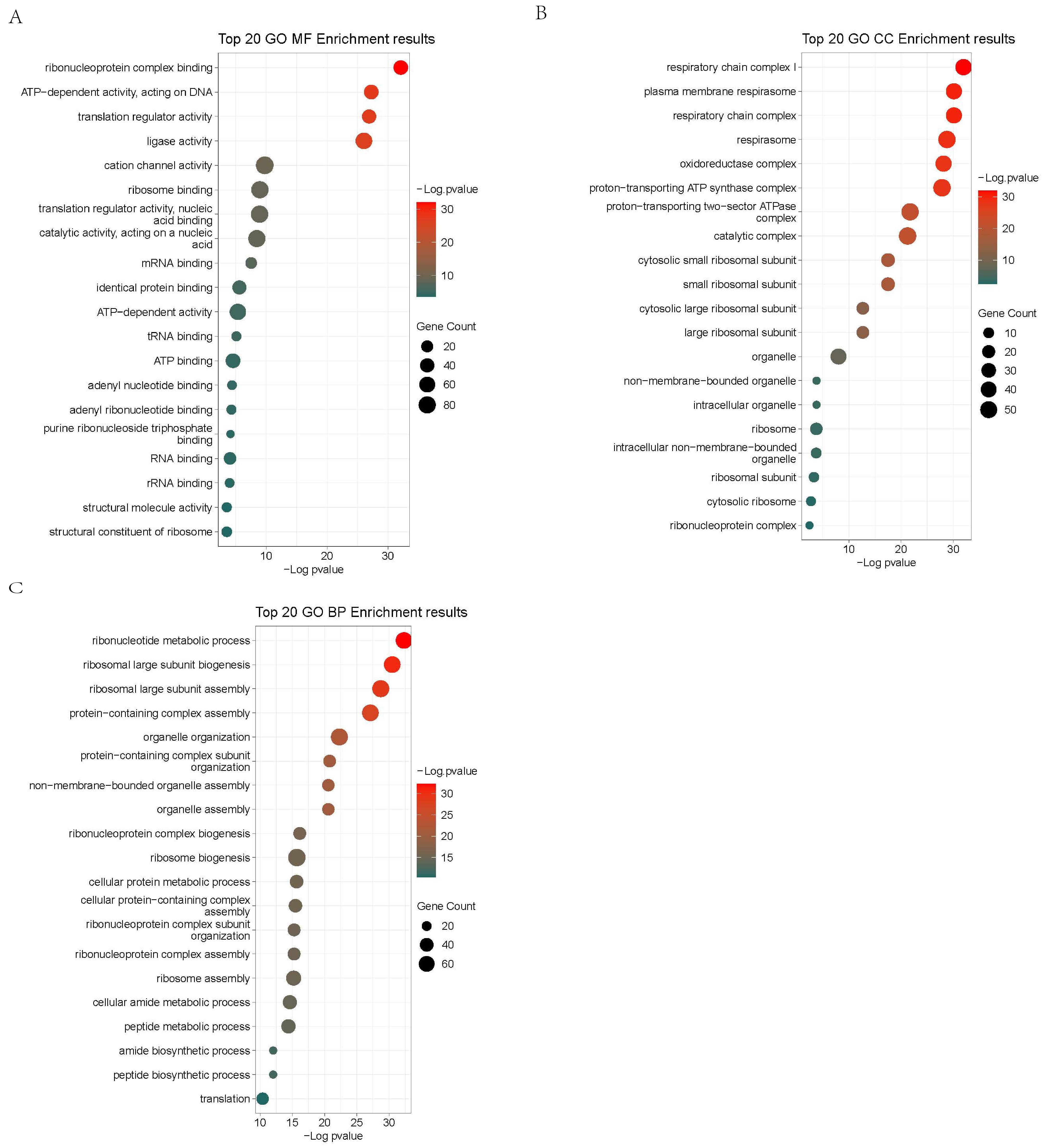

Supplement: Supplementary file 3 [file Image_2.JPEG]
