# Supplementary material for: Conjugation of plasmid harboring blaNDM-1 in a clinical Providencia rettgeri strain through the formation of a fusion plasmid
Source: Front Microbiol. 2023 Jan 4;13:1071385. doi: 10.3389/fmicb.2022.1071385 (PMC9845711; doi:10.3389/fmicb.2022.1071385)
Supplement: Supplementary file 1 [file Data_Sheet_1.zip › Table 2.DOC]

**TableS2:** 10 prophage regions have been identified, of which 4 regions are intact, 6 regions are incomplete, and 0 regions are questionable.

| **Region** | **Region Length** | **Completeness** | **Score** | **# Total Proteins** | **Region Position** | **Most Common Phage** | **GC %** |
| --- | --- | --- | --- | --- | --- | --- | --- |
|  |  |  |  |  | [18004577-genome](http://phaster.ca/submissions/ZZ_c3b09652c3#region_dna0) |  |  |
| 1 | 33.9Kb | incomplete | 40 | 40 | [20-34000](http://phaster.ca/submissions/ZZ_c3b09652c3#region_dna0) | PHAGE_Entero_Tyrion_NC_031077(23) | 42.33% |
| 2 | 43Kb | intact | 150 | 57 | [566459-609495](http://phaster.ca/submissions/ZZ_c3b09652c3#region_dna1) | PHAGE_Entero_mEp460_NC_019716(10) | 40.94% |
| 3 | 23.4Kb | incomplete | 30 | 20 | [1241490-1264905](http://phaster.ca/submissions/ZZ_c3b09652c3#region_dna2) | PHAGE_Entero_P4_NC_001609(3) | 41.66% |
| 4 | 55.2Kb | intact | 150 | 87 | [1260161-1315393](http://phaster.ca/submissions/ZZ_c3b09652c3#region_dna3) | PHAGE_Entero_ES18_NC_006949(15) | 42.21% |
| 5 | 33.7Kb | intact | 150 | 42 | [1446026-1479805](http://phaster.ca/submissions/ZZ_c3b09652c3#region_dna4) | PHAGE_Entero_Mu_NC_000929(25) | 46.16% |
| 6 | 22.1Kb | intact | 150 | 30 | [2702928-2725096](http://phaster.ca/submissions/ZZ_c3b09652c3#region_dna5) | PHAGE_Escher_500465_1_NC_049342(10) | 43.58% |
| 7 | 8.7Kb | incomplete | 40 | 9 | [3059994-3068784](http://phaster.ca/submissions/ZZ_c3b09652c3#region_dna6) | PHAGE_Salmon_SJ46_NC_031129(3) | 46.98% |
| 8 | 15Kb | incomplete | 30 | 14 | [4153083-4168135](http://phaster.ca/submissions/ZZ_c3b09652c3#region_dna7) | PHAGE_Escher_500465_1_NC_049342(7) | 38.71% |
|  |  |  |  |  | p[18004577](http://phaster.ca/submissions/ZZ_c3b09652c3#region_dna8)_NDM |  |  |
| 9 | 14.8Kb | incomplete | 20 | 26 | [218975-233789](http://phaster.ca/submissions/ZZ_c3b09652c3#region_dna8) | PHAGE_Salmon_SJ46_NC_031129(2) | 50.41% |
| 10 | 17.7Kb | incomplete | 30 | 15 | [235370-253130](http://phaster.ca/submissions/ZZ_c3b09652c3#region_dna9) | PHAGE_Salmon_SJ46_NC_031129(2) | 41.08% |

Intact (score > 90)
Questionable (score 70-90)
Incomplete (score < 70)

| **Region:** | The number assigned to the region. |
| --- | --- |
| **Region Length:** | The length of the sequence of that region (in bp). |
| **Completeness:** | A prediction of whether the region contains a intact or incomplete prophage based on the above criteria. |
| **Score:** | The score of the region based on the above criteria. |
| **# Total Proteins:** | The number of ORFs present in the region. |
| **Region Position:** | The start and end positions of the region on the bacterial chromosome. |
| **Most Common Phage:** | The phage(s) with the highest number of proteins most similar to those in the region. |
| **GC %:** | The percentage of GC nucleotides of the region. |
